# Influence of Forming Method on Cooked Characteristics of Ground Beef Patties

**DOI:** 10.3390/foods15132357

**Published:** 2026-07-02

**Authors:** Nina E. Gilmore, Autumn L. Armaly, Gabriela M. Bernardez-Morales, Savannah L. Douglas, Ricardo J. Barrazueta-Cordero, Sungeun Cho, Donald R. Mulvaney, Jason T. Sawyer

**Affiliations:** 1 Department of Animal Sciences, Auburn University, Auburn, AL 36849, USA; neg0027@auburn.edu (N.E.G.); ala0084@auburn.edu (A.L.A.); gzb0063@auburn.edu (G.M.B.-M.); sld0060@auburn.edu (S.L.D.); rzb0143@auburn.edu (R.J.B.-C.); mulvadr@auburn.edu (D.R.M.); 2 Department of Poultry Sciences, Auburn University, Auburn, AL 36849, USA; szc0181@auburn.edu

**Keywords:** Allo–Kramer shear force, cooked color, forming method, ground beef, sensory taste, tenderness

## Abstract

Consumer acceptability of whole-muscle and ground beef can largely be dictated by organoleptic properties. The objectives of the current study were to evaluate the organoleptic characteristics of ground beef patties manufactured using different forming attachments. Ground beef patties (*n* = 333/treatment) were randomly allotted to one of four treatments (Cavity, Nozzle, Manual, Guillotine) and subjected to analysis of cooking loss, cooking time, cooked patty shrinkage, internal cooked color, texture profile analysis, Allo–Kramer shear force, and consumer sensory panel. The forming method altered cooking time (*p* < 0.0001) and cooked patty shrinkage (*p* < 0.0001) but did not alter cooking loss (*p* = 0.8116). Instrumental hardness was greatest for beef patties formed using the Cavity method compared to all other forming treatments (*p* = 0.0002). The forming method did not alter internal cooked color redness (*p* > 0.5408), yellowness (*p* > 0.1053) nor red-to-brown (*p* > 0.4386). Lastly, consumer ratings for tenderness were altered by the forming method (*p* < 0.0020), but patty forming did not alter (*p* > 0.05) any other sensory characteristics. Categorizing the changes to the cooked characteristics of ground beef patties altered by forming and manufacturing techniques is important when determining consumer acceptance and quantifying textural differences. Current results indicate that ground beef patties formed using the nozzle method imparted the fewest detrimental changes to physiochemical and sensory traits when compared to all other forming methods. Nozzle-formed patties required the least amount of cooking time, the least instrumental resilience, the least instrumental force to shear, and the greatest perceived tenderness by consumer panelists.

## 1. Introduction

It is estimated that each day between 63 and 74% of people living in North America consume red meat [1]. In 2024, the average per capita beef consumption in the United States was reported to be at or near 26.8 kg per person [2]. Of the beef products consumed, ground beef is the most versatile menu option, making it a popular product among red meat consumers, and it can be commonly utilized in the form of a patty [3]. In the United States, ground beef comprised over 36% of fresh beef’s total dollar sales and 47% of the total volume (kg) of retail beef sold in 2024 [4]. Research has concluded that more than 50% of consumers report consuming at least one ground beef patty each week [5]. Additionally, in U.S. retail stores between January and July of 2021, 10.8%, or 73.5 million (kgs), of ground beef was sold as pre-formed patties [5]. Therefore, additional efforts in measuring and understanding the fresh or cooked meat characteristics of ground and formed beef products are necessary.

Palatability attributes of flavor, tenderness, and juiciness are considered the three most important factors contributing to overall liking of beef by consumers [6]. Many other factors have been documented over the past few decades highlighting the influence of breed type, intramuscular fat, moisture content, cooking method, or ingredient formulations. Survey results of university students (graduate and undergraduate) concluded that guaranteed tenderness label claims can significantly influence buying preferences of beef at the retail counter [7]. Changes in characteristics of beef steaks and patties have been well documented to be greatly influenced by cooking, fat content and moisture losses and are evaluated using objective measures such as Allo–Kramer shear force and Texture Profile Analysis [8].

It is well documented throughout the literature that many factors can influence the flavor profile of cooked meat proteins. Factors such as physiological maturity, gender, anatomical location of muscles, antemortem stressors, post-mortem chilling, intramuscular content, animal feeding, and gender are just a few topics investigators have recently focused on. More recently, processing implications post-mortem such as cooking, grinding, and processing aids, and manufacturing methodologies causing restructuring of muscle, can result in altering the meat protein flavor profile [9]. To evaluate changes in meat flavor an understanding of sensory profiles of meat can be subjectively and objectively evaluated by determining the taste profile of cooked meat products [10]. Traditionally, the sensory taste of cooked meat products has relied on the palate of meat consumers. Both quantitative and qualitative attributes of cooked meat have provided a greater understanding of the consumer demands for flavor. Currently, sensory consumer panels are being evaluated as a method to support subjective analysis with objective technology such as the electronic nose or electronic tongue. Unfortunately, these correlations remain under development [11] and the preferred method of capturing changes to cooked meat quality is sensory taste panels with human subjects.

One of the most important factors consumers consider when purchasing fresh and cooked beef is eating quality [12]. Meat flavor quality can be a subjective term that is defined by consumer experience in relation to the product. Consumers can determine flavor quality based on their experiences while eating. However, prior to consumption consumers rely on quality cues, which are any information stimuli perceived by the senses such as color or smell, to make determinations regarding products [13]. During the cooking process, meat characteristics including cooking shrink, cooking loss, cooked color and textural properties of beef are affected by protein denaturation [14,15]. Cooking shrink is a parameter that consumers oftentimes correlate with product quality, as greater amounts of cooking shrink are associated with decreased quality [16].

Formed beef patties eliminate the steps necessary to create a patty thus offering efficiency for consumers cooking at home and for restaurant establishments. Efficient ground beef patty cooking in restaurant settings is characterized by fast cooking times whereas consumers cooking ground beef patties at home seek a combination of efficiency and quality [17]. Previous research has been conducted investigating the relationship between various traits including fat percentage, grinding method, packaging type, and the sensory evaluation of ground beef [18,19,20]. However, limited research is available exploring the effect of the mechanical forming method on the sensory attributes and cooked characteristics of beef patties. Little is known about forming machine pressure settings, patty thickness, cavity depth, or knife cutting of weighted portions during the manufacturing of ground beef patties. Therefore, the objective of this study was to evaluate machine forming on the organoleptic properties and cooked parameters of ground beef patties.

## 2. Materials and Methods

### 2.1. Raw Materials

Beef chuck rolls (*n* = 32) were purchased from a commercial meat distributor (Capital Farms, Wickenburg, AZ, USA) and shipped using commercial refrigerated logistics to Robert Reiser & Co. (Canton, MA, USA). Within 5 days of carcass fabrication and packaging, chuck rolls were allotted randomly to one of four treatments (16.67 kg/batch, three batches/treatment) and each batch was coarse ground at the same time using a 3-knife bowl cutter (K124, Seydelmann, Stuttgart, Germany). Beef trimmings were chopped for 25 s using a knife speed of 1200 RPM and a bowl rotation speed of 15 RPM. Coarse ground beef was placed into a vacuum filler (HP25E, VEMAG, Verden, Germany) fitted with an in-line grinding attachment (982 In-Line Grinder, VEMAG, Verden, Germany) and a hard particle separator (813 separator, VEMAG, Verden, Germany). Following grinding, coarse ground was subdivided into batches and treatments prior to manufacturing into 118 g (±5 g) patties using one of four forming machines.

Beef patties were formed using one of four methods described in Table 1. Cavity-formed (Treatment 1) patties were manufactured using a VacForm (VF06, DJM Food Processing, Elburg, The Netherlands). A VacForm machine utilizes a waterwheel flow-dividing system that equally distributes the product across six cavities of the forming plate resulting in minimal product loss and weight variation [21]. Ground beef is released from the forming plate onto a conveyor using heated Teflon^®^-coated knock-out cups (Tomahawk Manufacturing, Inc., Plymouth, WI, USA) and can produce patties at a rate of up to 4000 kg/hour [22]. Nozzle-formed (Treatment 2) patties were formed using a Forming Machine (FM250, VEMAG, Verden, Germany). An FM250 patty former uses a rotating knife to cut weighted portions of ground beef fed through the nozzle resulting in a production speed of 1800 kg/hour [23]. Manual (Treatment 3) formed patties were produced using a Manual Patty Former (MPF818, VEMAG, Verden, Germany). A manual forming attachment connects directly to a vacuum filler allowing a machine operator to manually slide the forming plate for filling and forming; manufacturing capacity cannot exceed 540 kg/hour [24]. Guillotine (Treatment 4) patties were formed using a cut-off attachment (Robert Reiser & Co., Canton, MA, USA). Ground beef is fed through a 10.16 cm circular insert and is portioned using a Guillotine cut-off knife [25]. All patties, regardless of batch or treatment, passed under a flattening conveyor (FBC721, VEMAG, Verden, Germany) to achieve a target height of 1.6 cm and a target width of 10.1 cm. After forming, patties were crust-frozen in a blast freezer (QF080877 FBSM, Amerikooler, Hialeah, FL, USA) at −10 °C for two hours prior to individual packaging using a 125-micron forming film (T7050B, Sealed Air Corporation, Charlotte, NC, USA) and a 75-micron high-barrier non-forming film (T7230B, Sealed Air Corporation, Charlotte, NC, USA). A total of 1332 patties were packaged on a thermoforming vacuum packaging machine (RE20, Repak, Emmen, The Netherlands). Packaged patties were stored in a blast freezer (Model LHE6950, Larkin, Stone Mountain, GA, USA) at −22 °C (±2.1 °C) until laboratory analysis could be completed.

### 2.2. pH Value and Proximate Analysis

Beef patties (*n* = 6 patties × 3 batches × 4 treatments; *n* = 72 in total) were selected randomly during the entire manufacturing process of each batch and all treatments. Selected patties were individually packaged, identified, and used in the analysis of pH and proximate composition. Raw ground beef (2 g) was homogenized (Kinematica CH-6010, Brinkmann Instruments, Inc., Westbury, NY, USA) in 20 mL of deionized water inside a plastic centrifuge tube for 45 s. Homogenized patty pH was measured using a glass probe, temperature-compensating pH meter (Model-HI99163, Hanna Instruments, Woonsocket, RI, USA) and calibrated using 2-point standard buffers (Thermo Fisher Scientific, Chelmsford, MA, USA) at 4.0 and 7.0 pH values. Additionally, ground beef (160 g) was collected from all batches and all treatments were used to determine proximate analysis [26] with a near-infrared (NIR) spectrophotometer (Food ScanTM, FOSS Analytical A/S, Hilleroed, Denmark), and data were processed using ISIscanTM Software (version 4.8, Höganäs, Sweden). Patties from each batch across each treatment were analyzed for composition and reported as mean values for each treatment. Machine calibration is confirmed quarterly with an analysis of raw material and wet chemistry. Relative mean values for pH and proximate composition are included in Table 2.

### 2.3. Cooking Method

Prior to cooking, ground beef patties were thawed for 18 h in a refrigerator (Model 178GDC49HCB, Avantco Refrigeration, Alexandria, VA, USA) at 2.2 °C (±1.1 °C). Patties (*n* = 1 patty × 3 batches × 4 treatments; *n* = 12 in total) were placed on the cooking surface and cooked to an internal temperature of 73.9 °C on a commercial cooking griddle (Model HEG36E-1, Vulcan, Baltimore, MD, USA) preheated to 176.7 °C. Cooking runs (*n* = 20) were conducted across a single day for each analytical measure and to ensure consistent cooking, the internal temperature of each patty was measured using a digital cooking thermometer (Therma K-Plus, American Fork, UT, USA) placed in the geometric center of the patty. Patty location on the grill was recorded so that patties from all batches and all treatments were represented in each cooking run. Specifically, patties were assigned to grill locations so that all treatments and batches were randomly placed on the cooking surface. Patties were turned every 2 min during cooking to minimize crust formation and the cooking location of patties was rotated within the cooking run to minimize cooking variation [8].

### 2.4. Cooking Loss, Cooking Time and Shrinkage

Patties used for recording cooking loss (*n* = 20 patties × 3 batches × 4 treatments; *n* = 240 in total) were weighed prior to cooking on an analytical scale (Model PB3002-S, Mettler Toledo, Columbus, OH, USA). After cooking the patties to an internal temperature of 73.9 °C, the patties were cooled to room temperature and reweighed to record the final cooked weight. Cooking loss percentage was calculated using the following formula: [(raw weight—cooked weight) ÷ raw weight × 100%]. Ground beef patties were removed from their packages after thawing. The cooking time required to reach an internal temperature of 73.9 °C was recorded for each patty.

Patties used for measuring cook shrink were removed from their individual packages and two raw diameter measurements from each patty were recorded using calipers (Empire 2789, Empire Level, Mukwonago, WI, USA) prior to cooking. After cooking the patties to an internal temperature of 73.9 °C, the patties were cooled to room temperature and the cooked diameter was measured. Raw diameter and cooked diameter measurements were averaged for each patty. Cook shrink percentage was calculated using the following equation: [(average raw diameter—average cooked diameter) ÷ average raw diameter × 100%].

### 2.5. Instrumental Texture and Tenderness

Texture profile analysis (TPA) was performed on each patty (*n* = 20 patties × 3 batches × 4 treatments; *n* = 240 in total) following the AMSA guidelines [8] for meat cookery using a texture analyzer (Model TA-XTplus100C, Texture Technologies Corp., South Hamilton, MA, USA) and a 294.2 N load cell. From the center of each patty, 2.5 cm diameter samples were cut and underwent a two-cycle compression test. A 50 mm diameter cylindrical probe (TA-25A) was used to compress each patty sample to 70% of their initial height at a speed of 6.00 cm/min. Texture profile parameter values were collected for hardness (kg, peak force of the first compression); cohesiveness (behavior of a product under the second compression cycle compared to the first compression cycle); springiness (ability of a product to return to its original height following the first compression); chewiness (kg × cm, work necessary for mastication of the product (hardness × cohesiveness × springiness)); and resilience (recovery of the product following deformation relative to the speed and force endured during the first compression cycle), according to previously described procedures [27]. Shear force was measured in duplicate on a 2.5 cm wide strip from each patty with a 5-blade Allo–Kramer attachment (AKSF), using a speed of 3 mm/s and a load cell of 294.2 N. Maximum peak force was recorded during analysis in Newtons (N) according to methods outlined by the AMSA meat cookery guidelines [8].

### 2.6. Instrumental Cooked Color

The internal cooked color of patties (*n* = 20 patties × 3 batches × 4 treatments; *n* = 240 in total) was recorded using a HunterLab MiniScan EZ colorimeter (Model 45/0 LAV, Hunter Associates Laboratory Inc., Reston, WV, USA). Calibration of the colorimeter was performed according to the manufacturer’s guidelines using black and white color tiles. Cooked patties were sliced by hand using a knife horizontally through the geometric center of the patty. Three internal cooked color scans were taken to measure the objective lightness (L*), redness (a*), and yellowness (b*) of each patty using illuminant A, an aperture of 31.8 mm, and a 10° observer. Relative values for hue angle were calculated using the equation: tan^−1^ (b*/a*) [28]. Hue angle values indicate a shift from red to yellow. Relative values for chroma were calculated using the equation: √ (a*2 + b*2) [28]. Larger chroma values are indicative of a more vivid color. RTB (red to brown) values were calculated using a ratio of the reflectance values of 630 nm: 580 nm; values indicate a change from red to brown. Instrumental internal cooked color values were measured according to AMSA color guidelines [28].

### 2.7. Consumer Testing for Ground Beef Patties

Exempt use of human panelists was approved for this consumer sensory panel by the Auburn University Institutional Review Board (Exempt Protocol Number #STUDY00000126). Following approval, faculty, staff, and students from Auburn University who were 18 years or older and had a preference for consuming ground beef at least 3 to 4 times per month were recruited to participate in this consumer sensory panel. Consumer panelists (*n* = 93) evaluated samples of ground beef patties produced using four different forming methods. Serving order and sample presentation was randomly identified and balanced across all batches and treatments using RedJade software (version 6.1, Pleasant Hill, CA, USA). Sensory serving software assigned each treatment a random 3-digit code to be used during sensory analysis by consumer panelists. Ground beef patties (*n* = 8 patties × 3 batches × 4 treatments; *n* = 96 in total) were cooked to an internal temperature of 73.9 °C and cut into triangle samples (*n* = 4/patty) according to AMSA meat cookery guidelines [8]. Panelists evaluated one cooked patty triangle from each batch and treatment in isolated sensory booths under red-colored lighting to mask color variations between samples and reduce biases associated with cooked color perception. Unsalted saltine crackers (Nabisco, Mondelez Global LLC, East Hanover, NJ, USA) and room temperature water were provided to the panelists for palate cleansing between each sample. Overall liking and specific organoleptic properties (texture, aroma, appearance, flavor, juiciness, tenderness, beef flavor intensity, and perceived fattiness/leanness) were evaluated by consumer panelists on a 9-point hedonic scale (overall liking, texture, aroma, appearance, flavor: 1 = dislike extremely; 5 = neither like nor dislike; 9 = like extremely. Juiciness: 1 = extremely dry; 5 = neither dry nor juicy; 9 = extremely juicy. Tenderness: 1 = extremely tough; 5 = neither tough nor tender; 9 = extremely tender. Beef flavor intensity: 1 = none; 5 = neither bland nor intense; 9 = extremely intense. Perceived fattiness/leanness: 1 = extremely lean; 5 = neither lean nor fatty; 9 = extremely fatty). After the sample evaluation, panelists were asked to respond to a series of demographic questions, the results of which can be found in Table 3.

### 2.8. Statistical Analysis

Data were analyzed on version 9.4 of SAS^®^ (SAS Institute Inc., Cary, NC, USA) using the GLIMMIX model procedure as a randomized complete block design. Treatment was the fixed effect, and batch, panelist, and cooking run were included as the random effects in the statistical model. Least squares means were calculated for all independent variables. Pairwise *t*-tests were used to differentiate least squares means when significant *p*-values (*p* ≤ 0.05) were observed.

## 3. Results and Discussion

### 3.1. Cooking Loss, Cooking Time and Shrinkage

Cooking loss can be described as the change in product weight that occurs through the loss of water, fat, and protein during cooking [29,30]. Results of calculations for cooking losses of beef patties are presented in Table 4. Throughout the literature it is well discussed that factors including cooking method, fat content, and non-beef ingredients can alter cooking loss [31,32,33]. Previous research evaluating ground beef patties with varying fat percentages, cooked to two different endpoint temperatures, reported that increases in endpoint temperature and fat percentage caused cooking loss increases [18]. In contrast to the previously described study, fat percentages in the current study and endpoint cooking temperature remained consistent among the forming treatments. Results from the current study concluded that the patty-forming method (treatment) does not alter (*p* = 0.81) the cooking loss of the ground beef patties. It is plausible that the lack of changes to cooking loss among the treatments is attributed to the uniformity of raw materials, consistent cooking methods, and the absence of non-beef ingredients among the forming treatments. It is likely that the muscle fiber integrity was altered by the forming method due to the mechanical force applied to the product by each individual forming attachment. Previous research evaluating varying amounts of non-intact cells through inclusion percentages of a meat batter within ground beef patties can alter some characteristics. However, like the current study, those previous studies identified no significant interactions for cooking loss [34].

The cooking time of ground beef patties was altered (*p* < 0.0001) by the forming method in the current study (Table 4). Patties formed using a Guillotine required the greatest amount of cooking time (611.49 s) to reach an internal temperature of 73.9 °C while patties formed using the Nozzle and Manual attachments required the least amount of time (468.30 s and 471.79 s, respectively). Differences within individual patty manufacturing such as weight, thickness, cooking method and fat percentage can contribute to variations in cooking time [15,18,35]. Previous literature investigating these differences in cooked characteristics of patties formed using different filling methods suggested that decreased machine pressure produces a patty with less compaction and a less rigid structure allowing cooking surface heat to more quickly and easily penetrate the patty [36]. Prior research evaluating varying fat percentages in ground beef patties reported that increased fat percentages can lead to decreased cooking times, though an altered cook time of beef patties with fat levels between 10 and 25% was not significant [18]. Differences in cooking time among forming treatments in the current study may be attributed to the variation in structural compaction caused by the forming methods used. It is unlikely that the fat percentage of the ground beef patties altered cooking time in the current study as fat percentages ranged from 17.62 to 20.65% (Table 2).

Beef patty shrinkage because of cooking is a growing topic of concern throughout the meat industry in the U.S. Ground beef patties that shrink result in less coverage of a standardized patty bun which may lead to decreased consumer satisfaction [32]. Differences in the percentage of cooking shrinkage among the patties are presented in Table 4. There was an effect of forming treatment on the percentage of cooked shrink for the ground beef patties (*p* < 0.0001). Cooked patties formed using the Cavity and Manual methods had the greatest cooked percent shrinkage (>21%) whereas patties formed using a Guillotine cut-off recorded the least percent of cooked patty shrinkage (14.32%). Previous research investigating the effects of cooking on ground beef patty characteristics has reported that as internal patty temperature increases, the percent shrink of the patty also increases [14,29]. Ground meat products, such as beef patties, tend to have greater shrinkage due to a greater composition of whole muscle pieces resulting in a greater amount of muscle fibers present within the meat compared to an emulsified product, such as sausage, where a greater percentage of protein can be extracted during the chopping process [37]. It is plausible that the forming treatments altered the percentage of cooked patty shrink because of protein extraction caused by the mechanisms of the forming machines.

### 3.2. Instrumental Texture and Tenderness

Texture profile analysis (TPA) was conducted on the ground beef patties to obtain objective values for textural parameters (Table 5). Ground beef patties formed using a Cavity method were objectively harder (*p* = 0.0002) compared to patties formed using any other forming method. There were no differences (*p* = 0.9044) among forming methods for springiness. However, ground beef patties formed using the Cavity method had the greatest (*p* < 0.0001) cohesiveness, chewiness, and resilience values in the current study. Previous research highlights the inverse relationship between TPA values and the acceptability of a sample [38]. Research studies evaluating characteristics of ground beef patties have concluded that larger grinding plates and larger particle size of the ground meat can result in greater hardness and springiness instrumental values [39]. In addition, studies examining varying fat percentages of ground beef patties concluded that as fat percentage decreases, hardness and cohesiveness can increase [18,37]. It is well known and documented throughout the literature that the percentage of fat within a ground meat product provides a lubrication effect on objective measures of texture.

Allo–Kramer shear force (AKSF) was conducted to measure the instrumental tenderness of the cooked ground beef patties (Table 5). Greater AKSF values are associated with greater product toughness. Previous research examining the effects of mechanical processing on ground beef patty quality has concluded that smaller particle sizes and smaller grinder plate sizes used for processing ground meats can result in a reduction in instrumental tenderness [36,39]. Other well-documented factors including fat percentage and endpoint cooking temperature have also affected the instrumental tenderness of ground beef patties. A decline in the percentage of fat and a greater endpoint cooking temperature can increase instrumental shear force values [18,31,36,39]. In the current study there was an effect of forming treatment (*p* < 0.0001) for AKSF tenderness of the ground beef patties. Patties formed using the Nozzle method had the lowest AKSF values (*p* < 0.0001) compared to all other forming treatments. Consumer panelist ratings for tenderness agreed with instrumental measures that patties formed using the Nozzle, Manual and Guillotine attachments were more tender than patties formed using the Cavity method. Previous literature investigated the effect of patty formation pressure on break force [39]. Results concluded that ground beef patties with a 24% fat content manufactured using decreased amounts of pressure required a significantly lower break force when analyzed using AKSF [39]. Thus, it is plausible that the pressure conditions during the formation of patties using the Nozzle, Manual or Guillotine are much lower, resulting in less compaction of the ground beef and altering the instrumental tenderness of the patties. Current results confirm manufacturing of ground beef patties can cause a decline in shear force values.

### 3.3. Instrumental Cooked Color

Values for instrumental cooked color L*, a*, b*, chroma, hue angle, and RTB are presented in Table 6. Results from the current study indicate that patties formed using a Guillotine cut-off were darker (*p* < 0.0047) than patties from all other forming treatments (Table 6). Previous literature identifies a relationship between smaller particle sizes of meat and decreased lightness (L*) values [39]. As previously stated, it is possible that the Guillotine forming method caused greater amounts of protein extraction during patty formation which caused a reduction in meat particle size and thus decreased L* values. There was an effect of forming treatment (*p* < 0.0127) on the hue angle with patties formed using the Cavity method resulting in greater hue angle values compared to all other treatments. Previous literature examining the cooked color of ground beef patties with varying fat percentages when cooked to differing endpoint temperatures determined that a* and b* are not altered by fat percentage. However, patties with less fat and cooked to a greater internal end-point temperature have resulted in a darker internal cooked color [18]. Another study investigated the variability in the cooked color of beef patties under controlled cooking conditions and concluded that cooking time, cooking temperature, and internal temperature variations can alter internal cooked color properties of ground beef patties [40]. In the current study, internal endpoint temperature and fat percentage were consistent across all forming treatments. Results presented demonstrate that there were no significant differences for internal cooked color redness (a*; *p* = 0.541), yellowness (b*; *p* = 0.251), chroma (*p* = 0.657), or RTB (*p* = 0.439) among the forming treatments in the current study. Current results suggest that the forming method used to produce ground beef patties has a limited impact on the internal cooked color of the products.

### 3.4. Consumer Sensory Panel

Consumer panelists assigned hedonic and intensity ratings to the sensory attributes of ground beef patties formed using different machines (Table 7). No significant difference (*p* ≥ 0.0594) was observed for the overall liking, attribute liking (appearance, aroma, flavor, texture), perceived juiciness, beef flavor intensity, or perceived fattiness/leanness across the four treatments. Although there was no significant difference (*p* = 0.0594) detected amongst the treatments for juiciness, patties formed using the Nozzle received the greatest mean rating for juiciness followed by patties formed using the Guillotine. There was an effect of forming treatment (*p* < 0.0020) on tenderness identified by consumers. Cavity-formed patties were ranked significantly tougher by panelists compared to all other forming treatments. Interestingly, although the current results were not significant, numerically, consumers did rate patty differences for perceived fattiness/leanness between the ground beef patties. This perceived difference supports the hypothesis that the forming method can alter the organoleptic properties of ground beef patties considering all raw materials and processes leading up to the physical formation of the patty were identical. Previous literature highlights that mechanical forces, specifically during the grinding process, can affect meat quality [20]. Considering grinding procedures were consistent amongst the current forming treatments, it is plausible that the minimal detectable differences in the sensory attributes of the patties are due to varying mechanical forces acting on the meat during the formation of the patty.

## 4. Conclusions

Understanding the impact forming machines have on ground beef patty characteristics is necessary when ground beef remains an extremely popular protein choice for consumers in the United States. In the current study machinery used in forming ground beef patties did significantly alter organoleptic and meat quality attributes. While factors such as cooking loss, instrumental springiness, instrumental redness, yellowness, vividness, red to brown and sensory anchors of liking, appearance, aroma, flavor, texture, and beef flavor were not affected by the forming machine. Interestingly, guillotine patties required greater cooking time and had a darker internal cooked color. The forming method can alter cooking time, cooked patty shrinkage, instrumental tenderness, select internal color traits, and consumer-rated tenderness. Based on the current results it appears that less pressure during forming through the nozzle minimizes the deformation of cooked meat characteristics. However, ground beef patty manufacturers should consider the specific needs of their customers, manufacturing speed, and raw materials when identifying the most important meat attributes to achieve maximum consumer acceptability.

## Figures and Tables

**Table 1:** Treatment allocation and equipment specifications used during patty forming.

**Equipment**				
Treatment ^1^	Cavity	Nozzle	Manual	Guillotine
Forming	Set:	Nozzle:	Set: 10.16 cm	Insert 63.5 mm
Description	N01046.06.001	70 mm	Round	Round
Double screw	66C SS	48C × 72SC SS	48C × 72SC SS	48C × 72SC SS
**Operational Settings**				
Infeed	Continuous 1.1	Continuous 1.1	Pulsing 1.1	Pulsing 1.1
Pause	Single Portions	Continuous	1500 ms	300 ms
Speed	125 rpm	54.4 rpm	125 rpm	83.3 rpm
Target Weight	118 g	118 g	118 g	118 g
Program Weight *	765 g	104 g	108 g	107 g
Clip **	90 ms	50 ms	90 ms	120 ms
PPM	--	102	29.2	81.9

^1^ Treatments defined as: Cavity = DJM VF06 forming attachment manufactured by DJM Food Processing using a multi-cavity forming plate; Nozzle = VEMAG FM250 forming attachment manufactured by VEMAG using a rotating knife cutoff as weighted portions exit the nozzle; Manual = MPF818 forming attachment manufactured by VEMAG using a manual forming plate; Guillotine = Cutoff attachment manufactured by VEMAG with a knife cutoff of weighted portions; * Program weight is defined as the target weight specification of the forming machine based on the forming cavity. ** Knife speed in milliseconds.

**Table 2:** Mean values for pH and proximate analysis of beef patties.

Treatment ^1^	Cavity	Nozzle	Manual	Guillotine
pH	6.04	5.99	6.00	6.01
Moisture (%)	69.79	66.20	63.88	66.98
Protein (%)	19.89	20.86	22.16	21.55
Fat (%)	17.62	20.14	20.65	18.58

^1^ Treatment defined as: Cavity = DJM VF06 forming attachment manufactured by DJM Food Processing using a multi-cavity forming plate; Nozzle = VEMAG FM250 forming attachment manufactured by VEMAG using a rotating knife cutoff as weighted portions exit the nozzle; Manual = MPF818 forming attachment manufactured by VEMAG using a manual forming plate; Guillotine = Cutoff attachment manufactured by VEMAG with a knife cutoff of weighted portions.

**Table 3:** Consumer panel demographic frequencies.

	Respondents	Percentage
Sex		
Male	47	50.54%
Female	46	49.46%
Age		
18 to 20 years	4	4.30%
21 to 29 years	56	60.22%
30 to 39 years	20	21.50%
40 to 49 years	10	10.72%
50 to 59 years	1	1.08%
60 to 69 years	2	2.15%
Highest level of education achieved		
High School Diploma/GED	7	7.53%
2-year college degree	1	1.08%
4-year college degree	40	43.01%
Graduate degree (Master’s, Doctorate, etc.)	45	48.39%
Ethnicity		
Caucasian	43	46.24%
Latino/Hispanic	25	26.88%
Asian or Pacific Islander	13	13.98%
African American	12	12.90%
Income		
Less than USD 30,000	64	68.82%
USD 30,000 to USD 49,999	9	9.68%
USD 50,000 to USD 79,999	12	12.90%
Greater than USD 80,000	8	8.60%
Consumes Beef Patties		
Once a day or more	2	2.15%
More than 3 times per week	10	10.75%
2 to 3 times per week	24	25.81%
Once a week	23	24.73%
2 to 3 times per month	26	27.96%
Once a month	5	5.38%
Less than once a month	3	3.23%
Purchasing Beef Patties		
Once a week	15	16.13%
Once every 2 or 3 weeks	42	45.16%
Once a month	21	22.58%
Once every 2 to 3 months	12	12.90%
Once every 4 to 6 months	1	1.08%
Less than once a year	2	2.15%

**Table 4:** Influence of forming method on cooked beef patties.

Patty Forming Treatment ^1^						
	Cavity	Nozzle	Manual	Guillotine	SEM *	*p*-Value
Cook Loss (%) ^2^	41.9	42.1	42.1	41.8	0.38	0.812
Cook Time (s) ^3^	559.6 ^b^	468.3 ^c^	471.8 ^c^	611.5 ^a^	8.40	<0.0001
Cook Shrink (%) ^4^	22.2 ^a^	18.3 ^b^	21.3 ^a^	14.3 ^c^	0.57	<0.0001

^1^ defined as: Cavity = DJM VF06 forming attachment manufactured by DJM Food Processing using a multi-cavity forming plate; Nozzle = VEMAG FM250 forming attachment manufactured by VEMAG using a rotating knife cutoff as weighted portions exit the nozzle; Manual = MPF818 forming attachment manufactured by VEMAG using a manual forming plate; Guillotine = Cutoff attachment manufactured by VEMAG with a knife cutoff of weighted portions. ^2^ Cook loss is expressed as a percentage (%). ^3^ Cook time is reported in seconds (Cook Time (s)). ^4^ Cook shrink is expressed as a percentage (%). ^a–c^ Mean values within a row lacking common superscripts differ (*p* < 0.05). * SEM, standard error of the mean.

**Table 5:** Instrumental textural measurement of ground beef patties produced using different forming attachments.

Patty Forming Treatment ^1^						
	Cavity	Nozzle	Manual	Guillotine	SEM *	*p*-Value
Hardness (kg) ^1^	2.6 ^a^	2.3 ^b^	2.2 ^b^	2.2 ^b^	0.08	0.0002
Springiness ^2^	0.73	0.74	0.74	0.74	0.007	0.9044
Cohesiveness ^3^	0.48 ^a^	0.42 ^c^	0.46 ^b^	0.43 ^c^	0.005	<0.0001
Chewiness ^4^	0.94 ^a^	0.74 ^b^	0.76 ^b^	0.70 ^b^	0.031	<0.0001
Resilience ^5^	0.19 ^a^	0.16 ^d^	0.17 ^b^	0.17 ^c^	0.002	<0.0001
ASKF (N) ^6^	286.5 ^a^	148.7 ^d^	214.6 ^c^	225.3 ^b^	3.65	<0.0001

^1^ Treatment defined as: Cavity = DJM VF06 forming attachment manufactured by DJM Food Processing using a multi-cavity forming plate; Nozzle = VEMAG FM250 forming attachment manufactured by VEMAG using a rotating knife cutoff as weighted portions exit the nozzle; Manual = MPF818 forming attachment manufactured by VEMAG using a manual forming plate; Guillotine = Cutoff attachment manufactured by VEMAG with a knife cutoff of weighted portions. ^2^ Springiness values are a ratio of the time duration of the second compression to that of the first compression (duration 2/duration 1). ^3^ Cohesiveness values are a ratio of the positive force area during the second compression to that during the first compression (area 2/area 1). ^4^ Chewiness values = hardness × cohesiveness × springiness. ^5^ Resilience values are a ratio of the first cycle decompression area to the first cycle compression area (area 1b/area 1a). ^6^ Allo–Kramer shear force is reported in Newtons (N). ^a–d^ Mean values within a row lacking common superscripts differ (*p* < 0.05). * SEM, standard error of the mean.

**Table 6:** Effect of forming on instrumental cooked color of ground beef patties.

Patty Forming Treatment ^1^						
	Cavity	Nozzle	Manual	Guillotine	SEM *	*p*-Value
L* ^2^	53.9 ^a^	54.3 ^a^	53.9 ^a^	52.5 ^b^	0.37	0.005
a* ^3^	16.1	16.8	16.6	16.3	0.36	0.541
b* ^4^	20.1	19.6	19.2	19.4	0.23	0.251
Hue Angle ^5^	51.4 ^a^	49.9 ^b^	49.5 ^b^	50.1 ^b^	0.43	0.013
Chroma ^6^	25.8	25.9	25.4	25.4	0.38	0.657
RTB ^7^	1.78	1.90	1.86	1.81	0.06	0.439

^1^ Treatments defined as: Cavity = DJM VF06 forming attachment manufactured by DJM Food Processing using a multi-cavity forming plate; Nozzle = VEMAG FM250 forming attachment manufactured by VEMAG using a rotating knife cutoff as weighted portions exit the nozzle; Manual = MPF818 forming attachment manufactured by VEMAG using a manual forming plate; Guillotine = Cutoff attachment manufactured by VEMAG with a knife cutoff of weighted portions. ^2^ L* (lightness) values are a measure of darkness to lightness; larger values indicate a lighter color (0 = black, 100 = white). ^3^ a* (redness) values are a measure of redness; larger values indicate a redder color (−60 = green, +60 = red). ^4^ b* (yellowness) values, larger values indicate a yellower color (−60 = blue, +60 = yellow). ^5^ Hue angle values represent the change from the true red axis; larger values indicate a greater shift from red to yellow. ^6^ Chroma is a measure of the total color; larger values indicate more total color. ^7^ RTB (Red to Brown) is a ratio of 630 nm:580 nm that represents a change in color from red to brown; larger values indicate a redder color. ^a,b^ Mean values within a row lacking common superscripts differ (*p* < 0.05). * SEM, standard error of the mean.

**Table 7:** Consumer panelist ratings of ground beef patties produced using different forming attachments.

Patty Forming Treatment ^1^						
Sensory Anchor ^2^	Cavity	Nozzle	Manual	Guillotine	SEM *	*p*-Value
Overall Liking	6.4	6.5	6.3	6.7	0.18	0.257
Appearance	6.9	6.8	6.6	6.9	0.17	0.237
Aroma	6.9	6.9	6.9	7.1	0.15	0.663
Flavor	6.6	6.6	6.4	6.5	0.19	0.896
Texture	6.3	6.3	6.2	6.5	0.21	0.678
Juiciness	6.1	6.7	6.3	6.5	0.18	0.060
Tenderness	5.8 ^b^	6.6 ^a^	6.4 ^a^	6.3 ^a^	0.18	0.002
Beef Flavor Intensity	5.9	5.8	5.9	6.1	0.18	0.532
Perceived Fattiness/Leanness	5.5	5.9	6.1	5.8	0.17	0.100

^1^ Treatment defined as: Cavity = DJM VF06 forming attachment manufactured by DJM Food Processing using a multi-cavity forming plate; Nozzle = VEMAG FM250 forming attachment manufactured by VEMAG using a rotating knife cutoff as weighted portions exit the nozzle; Manual = MPF818 forming attachment manufactured by VEMAG using a manual forming plate; Guillotine = Cutoff attachment manufactured by VEMAG with a knife cutoff of weighted portions. ^2^ Sensory anchors: Consumer panelists evaluated each sensory anchor using a 9-point hedonic scale (1 = dislike extremely, 9 = like extremely). ^a,b^ Mean values within a row lacking common superscripts differ (*p* < 0.05). * SEM, standard error of the mean.

## Data Availability

The original contributions presented in this study are included in this article; further inquiries can be directed to the corresponding author.

## Abbreviations

The following abbreviations are used in this manuscript:

AKSF	Allo–Kramer shear force
TPA	Texture profile analysis
AMSA	American Meat Science Association
SEM	Standard Error of the Mean
